# HKAN: A Hybrid Kolmogorov–Arnold Network for Robust Fabric Defect Segmentation

**DOI:** 10.3390/s24248181

**Published:** 2024-12-21

**Authors:** Min Li, Pei Ye, Shuqin Cui, Ping Zhu, Junping Liu

**Affiliations:** School of Computer and Artificial Intelligence, Wuhan Textile Unversity, Wuhan 430200, China; 2008031@wtu.edu.cn (M.L.); csq@wtu.edu.cn (S.C.); zhuping@wtu.edu.cn (P.Z.); jpliu@wtu.edu.cn (J.L.)

**Keywords:** fabric defect detection, CNN, transformer, KANs, semantic segmentation

## Abstract

Currently, fabric defect detection methods predominantly rely on CNN models. However, due to the inherent limitations of CNNs, such models struggle to capture long-distance dependencies in images and fail to accurately detect complex defect features. While Transformers excel at modeling long-range dependencies, their quadratic computational complexity poses significant challenges. To address these issues, we propose combining CNNs with Transformers and introduce Kolmogorov–Arnold Networks (KANs) to enhance feature extraction capabilities. Specifically, we designed a novel network for fabric defect segmentation, named HKAN, consisting of three components: encoder, bottleneck, and decoder. First, we developed a simple yet effective KANConv Block using KAN convolutions. Next, we replaced the MLP in PoolFormer with KAN, creating a lightweight KANTransformer Block. Finally, we unified the KANConv Block and the KANTransformer Block into a Hybrid KAN Block, which serves as both the encoder and bottleneck of HKAN. Extensive experiments on three fabric datasets demonstrate that HKAN outperforms mainstream semantic segmentation models, achieving superior segmentation performance and delivering prominent results across diverse fabric images.

## 1. Introduction

Fabric defect detection is a quality control process that employs various technical methods to inspect textiles either during production or after the completion of finished products [1]. Common defects include issues such as uneven coloration, inconsistent texture, structural damages (e.g., yarn breakage, holes, and cracks), surface imperfections (e.g., oil stains and wrinkles), and edge problems like bad selvedge and color discrepancies [2]. The goal of fabric defect detection is to ensure that textiles meet quality standards, minimize the presence of defective products in the market, and enhance the overall competitiveness of the products.

**Figure 1:**
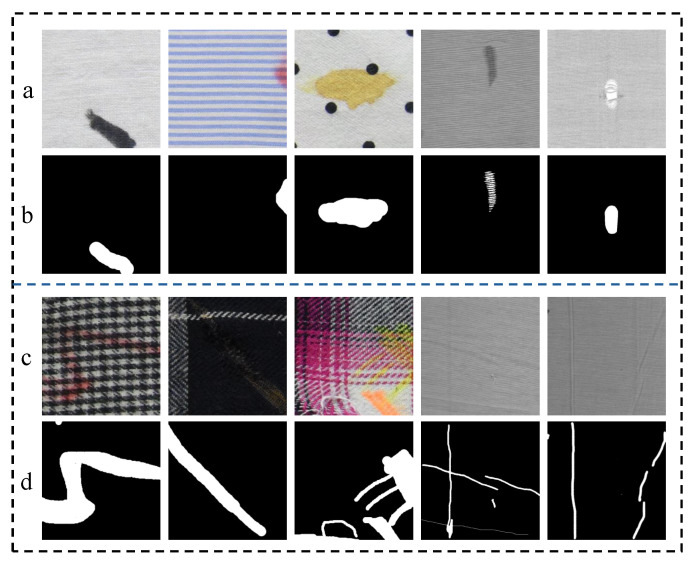
Example of fabric defects. (**a**,**c**) are defects in simple and complex textured fabrics, respectively, while (**b**,**d**) are their corresponding ground truth.

In the early textile manufacturing industry, fabric defects were primarily identified through manual visual inspection [3]. However, with advancements in industrial automation, this labor-intensive and time-consuming approach has been gradually replaced. In recent years, deep learning, particularly convolutional neural networks (CNNs), has revolutionized computer vision and provided new solutions for fabric defect detection. For instance, Zhao et al. [4] proposed MSCNN, which achieves efficient and accurate real-time defect detection by capturing multi-scale feature maps and incorporating K-means clustering analysis. Similarly, Huang et al. [5] combined segmentation and decision networks to deliver high-precision, real-time defect detection.Beyond standard CNN applications, some researchers have explored integrating domain-specific knowledge to improve detection. Koulali et al. [6] introduced an unsupervised learning framework with dynamic feature selection to simplify training and enhance detection accuracy. Chen et al. [7] improved Faster R-CNN [8] by incorporating Gabor filters into its first convolutional layer, effectively reducing interference from background textures, and used a genetic algorithm to optimize filter parameters. Lu et al. [9] proposed TADet, a texture-aware, one-stage detection network that leverages adaptive feature fusion and multi-task training, addressing the limitations of traditional one-stage approaches by incorporating crucial texture information. While these CNN-based methods demonstrate strong performance in detecting simple, localized defects (e.g., Figure 1a), they struggle with modeling long-range dependencies. This limitation becomes evident when defects span multiple regions or involve complex background textures, as shown in Figure 1c, where performance significantly degrades.

Another notable model in deep learning is Transformer [10], based on a self-attention mechanism, which effectively captures long-range dependencies between sequences and has achieved remarkable results in natural language processing. Vision Transformer (ViT) [11] is the first to apply the Transformer architecture to computer vision, yielding impressive results in image classification tasks. Following this, Swin Transformer [12] introduced a hierarchical structure and a more efficient self-attention mechanism, achieving state-of-the-art (SOTA) results across various downstream computer vision tasks, marking a significant milestone in the field. Inspired by these advancements, some researchers have also begun to explore the use of Transformers for fabric defect detection. Qu et al. [13] proposed a novel network called U-SMR for fabric defect detection. U-SMR integrates ResNet-50 [14] with Swin Transformer modules, combining global contextual features, defect detail features, and high-level semantic features, achieving excellent performance on the ZJU-Leaper dataset [3]. Xu et al. [15] introduced a fabric defect segmentation model based on an improved Swin-Unet [16] and Gabor filters. Specifically, the model incorporates Gabor filters to extract low-level texture features, reducing texture interference and accelerating convergence during the early training stages. Additionally, a non-fully symmetric U-shaped architecture is designed to simplify model training, while multi-stage result fusion is employed to achieve precise defect localization.

Regardless of whether they are CNNs or Transformers, an indispensable component of these models is the Multi-Layer Perceptrons (MLPs) [17]. Serving as a core building block of neural networks, MLPs play a crucial role in various deep learning architectures due to their strong capability for nonlinear fitting. Recently, the emergence of Kolmogorov–Arnold Networks (KANs) [18] has demonstrated the potential to address this issue. KANs enhance model interpretability by introducing learnable activation functions on the edges rather than at the nodes, providing a novel perspective. Moreover, by replacing weight parameters with learnable functions on the edges, KANs utilize model parameters more efficiently, thereby reducing the computational burden of the model. In this paper, we aim to explore the potential of integrating KANs with CNNs and Transformers, applying this combination to the field of fabric defect segmentation. Our main contributions are as follows:

- We constructed a Hybrid KAN model that integrates CNNs, Transformers, and KANs into a unified framework, leveraging the strengths of each component. CNNs are used to extract local features, Transformers capture global dependencies, while KANs accurately capture complex patterns and subtle differences in fabric defect images through their flexible nonlinear feature extraction capabilities.
- We proposed a simple KAN Conv Block (KCB) using KANConv to extract local features from images. Compared to traditional convolutional modules, KCB reduces model complexity and parameter count while maintaining similar accuracy.
- We replaced the traditional MLP in the Transformer architecture with KAN, constructing a new KAN Transformer Block (KTB) for extracting global features from images. KTB leverages the characteristics of KAN to enhance the Transformer’s ability to capture global contextual information in images, making it more adaptable when dealing with complex fabric texture images.

## 2. Related Work

### 2.1. Semantic Segmentation

The foundational work in semantic segmentation, FCN [19], achieved pixel-level prediction by replacing fully connected layers with convolutional layers. U-Net [20] further advanced this field by introducing an encoder-decoder structure that effectively combines low-level and high-level features through skip connections, making it one of the most widely used architectures across diverse applications. Subsequently, CNN-based methods [21,22,23,24,25] have pushed the boundaries of semantic segmentation by expanding receptive fields, integrating multi-scale information, and employing feature fusion techniques. Building on the success of Transformers in natural language processing, their powerful global modeling capabilities have been adapted for semantic segmentation [26,27,28,29,30,31]. Transformer-based models have introduced self-attention mechanisms and global context modeling, significantly improving segmentation accuracy and adaptability to complex scenes. The method proposed in this paper combines the complementary strengths of CNNs and Transformers for feature extraction, aiming to achieve more comprehensive and precise segmentation of fabric defects by leveraging the local feature modeling of CNNs and the global context capture of Transformers.

### 2.2. Kolmogorov–Arnold Networks

Recently, Kolmogorov–Arnold Networks (KANs) have emerged as a novel architecture that improves interpretability and accuracy over traditional multilayer perceptrons (MLPs). Researchers have increasingly applied KANs to various computer vision tasks. For example, U-KAN [32] enhances the encoder-decoder structure of U-Net [20] by integrating KAN layers with nonlinear activation functions, boosting feature extraction and improving model accuracy, efficiency, and interpretability. Cambrin et al. [33] applied U-KAN to crop area segmentation and used post-hoc explanation techniques to evaluate saliency maps, highlighting the distinct visual regions emphasized by U-KAN and U-Net during predictions. Similarly, Bodner et al. [34] incorporated KANs’ nonlinear activation functions into convolutional layers, creating Convolutional KANs that achieve superior parameter efficiency and offer a novel approach to neural network optimization. In addition, Yang et al. [35] proposed the Kolmogorov–Arnold Transformer (KAT), replacing B-Spline functions with rational functions to accelerate computations and substituting MLP layers with Group-Rational KANs within the Transformer architecture. Experiments demonstrate that KAT outperforms MLP-based Transformers across various vision tasks while maintaining similar computational complexity. Building on this, He et al. [36] introduced MLP-KAN, which combines MLPs and KANs under the Mixture-of-Experts (MoE) framework. This design unifies deep representation learning and function learning, allowing MLP-KAN to dynamically adapt to task characteristics. As a result, MLP-KAN demonstrates superior flexibility and efficiency across diverse domains, optimizing performance based on task-specific needs.

## 3. Methodlogy

In this section, we first give a brief introduction to KAN, then present an overview of our proposed approach, and finally the proposed hybrid KAN block will be presented in detail.

### 3.1. Preliminary

MLPs are one of the fundamental components of current deep learning models, renowned for their ability to approximate complex nonlinear functions through the universal approximation theorem. However, MLPs are not perfect nonlinear regressors. As the number of layers in the network deepens, the internal workings of MLPs become less interpretable, failing to meet the demands for transparency and interpretability in practical applications. Additionally, MLPs can be parameter-heavy, especially in models like Transformers where they dominate the parameter count, leading to efficiency concerns.

As a novel neural network architecture, KANs demonstrate the potential to replace MLPs. KANs are rooted in the Kolmogorov–Arnold representation theorem, which allows for the representation of multivariate functions as compositions of univariate functions and additions. Unlike MLPs, which employ fixed activation functions at the nodes, KANs place learnable activation functions on the edges, parameterized as splines. This innovative design eschews linear weights altogether, with each weight replaced by a learnable univariate function. Compared to MLPs, KANs have higher transparency and interpretability, and they exhibit faster neural scaling laws, allowing them to achieve greater accuracy with fewer parameters. KANs are defined by their core equation, which is an embodiment of the Kolmogorov–Arnold representation theorem. For a function $f(x_{1},\ldots ,x_{n})$ defined on a bounded domain, it can be expressed as

(1) $$f\left(x\right)=f\left(x_{1},\cdots ,x_{n}\right)=\sum_{q=1}^{2n+1}\Phi_{q}\left(\sum_{p=1}^{n}\phi_{q,p}\left(x_{p}\right)\right)$$

where $\phi_{q,p}$ and $\Phi_{q}$ are continuous univariate functions. This equation is the linchpin of KANs, allowing them to decompose complex functions into a series of simpler, learnable components. This decomposition is not only mathematically elegant but also provides a foundation for KANs’ enhanced accuracy and interpretability, as it leverages the compositional nature of functions more effectively than MLPs.

For an input vector x, the output of the KAN is given by

(2) $$\mathrm{KAN}\left(x\right)=\left(\Phi_{L-1}\circ \Phi_{L-2}\circ \cdots \circ \Phi_{1}\circ \Phi_{0}\right)x$$

where $\Phi_{i}$ represents the set of univariate functions (splines) that are applied to the inputs from the previous layer. The composition ∘ indicates that the output of one function set is used as the input to the next.

In summary, while MLPs use fixed activation functions and linear combinations of inputs, the KANs use learnable activation functions that are applied to each input separately and are composed in a multi-layer structure. This allows KANs to capture more complex and compositional structures in the data.

### 3.2. Overview of the Proposed Method

**Figure 2:**
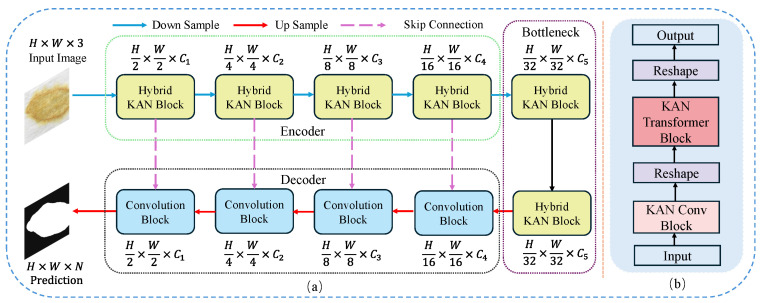
Overview of the HAKN framework. (**a**) The overall architecture of HKAN consists of three parts: encoder, bottleneck, and decoder. The encoder and bottleneck are composed of Hybird KAN Blocks, while the decoder utilizes a Convolution Block. (**b**) Detailed illustration of the Hybird KAN Block, primarily consisting of KAN Conv Block and KAN Transformer Block.

The framework of the method proposed in this paper is shown in Figure 2a. It is designed similarly to U-Net, consisting of encoder, bottleneck, and decoder. The encoder and bottlenetck are composed of multiple cascaded Hybrid KAN Blocks, while the decoder comprises several convolutional blocks. Notably, the Hybrid KAN Block is a novel module we designed, which integrates KAN with CNN and Transformer into a unified framework, as illustrated in Figure 2b.

Specifically, for an input image $I\in R^{H\times W\times C}$ where H, W, and C represent the height, width, and number of channels of the image, respectively. Initially, the input image *I* is fed into a four-stage encoder to extract features at different levels. During this process, the size of the feature maps sequentially reduces to {1/2, 1/4, 1/8, 1/16} of the original image dimensions. The encoder captures progressively more abstract features of the input as the dimensions decrease. In addition, the network incorporates a skip-connection mechanism that helps retain spatial information from higher-resolution feature maps by directly connecting layers of similar dimensions. After the encoding phase, the bottleneck layer aggregates the features in order to capture the most critical information, setting the stage for effective feature reconstruction. The upsampling process then initiates, where each upsampling step is paired with convolutional layers to meticulously refine the spatial details of the reconstructed output.

### 3.3. Hybrid KAN Block

Our proposed Hybrid KAN Block is a brand-new module that integrates KAN into the CNN and Transformer frameworks, forming a unified whole, as shown in Figure 2b. Let the input feature map be denoted as $X\in R^{H\times W\times C}$, the input feature map *X* first passes through the KAN Convolutional Block:

(3) $$X_{conv}=\mathrm{KANConv}\left(X\right)$$

where $X_{conv}\in R^{H\times W\times 2C}$. The output from the KAN Convolutional Block, $X_{conv}$ is then reshaped to fit the input dimensions required by the Transformer block:

(4) $$X_{reshaped}=\mathrm{Reshape}\left(X_{conv}\right)$$

where $X_{reshaped}\in R^{N\times 2C}$ and N=H×W. This reshaped output is then fed into the KAN Transformer Block to capture long-range dependencies within the feature map:

(5) $$X_{transformed}=\mathrm{KANTransformer}\left(X_{reshaped}\right)$$

The output from the Transformer block, $X_{transformed}$, is reshaped back to spatial dimensions:

(6) $$X_{out}=\mathrm{Reshape}\left(X_{transformed}\right)$$

where $X_{out}\in R^{H\times W\times 2C}$. The final output $X_{out}$ is the processed feature map from the Hybrid KAN Block, which integrates both CNN-based local feature extraction and Transformer-based global feature modeling.

#### 3.3.1. KAN Conv Block

KAN Convolutions [34] represent an innovative approach to traditional convolutional operations within neural networks, drawing inspiration from the Kolmogorov–Arnold theorem. Unlike typical convolutional networks that utilize fixed activation functions, KAN Convolution employs B-Spline functions which are defined as a linear combination of basis splines. Each element within the convolutional kernel is a B-Spline function:

(7) $$\phi \left(x\right)=\sum_{i}c_{i}B_{i}\left(x\right)$$

Here, $B_{i}\left(x\right)$ are basis spline functions, and $c_{i}$ are trainable coefficients. These coefficients are adjusted during training, allowing the convolutional layer to mold its activation functions according to the learned data patterns. In KAN Convolution, the application of the kernel to the input feature map involves a unique adaptive process where each kernel element has its own B-Spline activation function. The output feature map at each location is computed as

(8) $${\left(Image*K\right)}_{i,j}=\sum_{k=1}^{N}\sum_{l=1}^{M}\phi_{kl}\left(Image_{i+k,j+l}\right)$$

here, *K* is a KAN convolution kernel of size N×M, $\phi_{kl}$ are the B-Spline activation functions applied uniquely at each kernel position (k,l), enriching the convolution operation with the ability to capture a wider range of features through these adaptive nonlinear transformations.

To handle inputs outside the predefined grid range, the grid is dynamically extended. This is formulated as an optimization problem to adjust the control points $c_{j}^{\prime }$ of the B-Splines:

(9) $$\left\{c_{j}^{\prime }\right\}=\arg\min_{c_{j}^{\prime }}E_{x∼p(x)}\left[\sum_{j=0}^{G2+k-1}c_{j}^{\prime }B_{j}^{\prime }\left(x^{\prime }\right)-\sum_{j=0}^{G1+k-1}c_{j}B_{j}\left(x\right)\right]\;$$

where G1 is the previous grid size, G2 is the new grid size, and *k* is the degree of the B-Spline.

**Figure 3:**
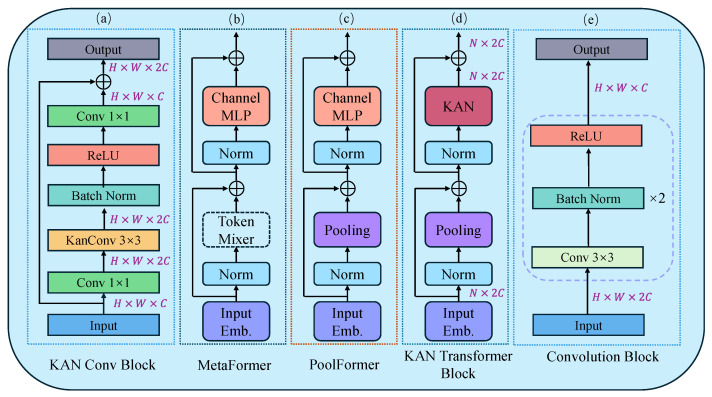
Specific architecture of each module. (**a**) KAN Conv Block. (**b**) MetaFormer. (**c**) PoolFormer. (**d**) KAN Transformer Block. (**e**) Convlution Block. The KAN Conv Block and KAN Transformer Block are components of the Hybrid KAN Block, and the Convolution Block serves as the decoder.

The overview of our proposed KAN Conv Block is illustrated in Figure 3a. The input feature map $F_{in}$ first undergoes a 1×1 convolution to double the depth of the feature map while maintaining its spatial dimension. After the initial convolution, the feature map $F_{1}$ is subjected to a specialized KAN convolution with a 3×3 kernel to extract key spatial features. The resulting feature map $F_{2}$ is then normalized using batch normalization to stabilize the learning process. The batch-normalized feature map $F_{3}$ is subsequently passed through a Rectified Linear Unit (ReLU) activation function to mitigate the gradient vanishing problem. The activated feature map $F_{4}$ is processed through another 1×1 convolution to refine the feature map. Finally, the output feature map $F_{out}$ is element-wise added with the original input $F_{in}$ through a skip connection, ensuring that the block contributes enhancements without losing original features. The whole process of KAN Conv Block can be expressed as follows:

(10) $$F_{1}={\mathrm{Conv}}_{1\times 1}\left(F_{in}\right)$$

(11) $$F_{2}={\mathrm{KanConv}}_{3\times 3}\left(F_{1}\right)$$

(12) $$F_{3}=\mathrm{BatchNorm}\left(F_{2}\right)$$

(13) $$F_{4}=\mathrm{ReLU}\left(F_{3}\right)$$

(14) $$F_{5}={\mathrm{Conv}}_{1\times 1}\left(F_{4}\right)$$

(15) $$F_{out}=F_{in}+F_{5}$$

where $F_{in},F_{5}\in R^{H\times W\times C}$ and $F_{1},F_{2},F_{3},F_{4},F_{out}\in R^{H\times W\times 2C}$.

#### 3.3.2. KAN Transformer Block

The Transformer architecture is highly regarded in the field of deep learning due to its ability to capture long-range dependencies. The emergence of Vision Transformer [11] and Swin Transformer [12] has also highlighted the potential for Transformer technology in the field of computer vision. PoolFormer [37] builds on the Transformer by distilling a general architecture called MetaFormer, and it utilizes simple pooling operations as a token mixer, which improves computational efficiency while maintaining strong performance. As seen in Figure 3, the MLP is an indispensable component of the Transformer architecture, used to enhance the model’s capability to process non-linearities. Although the MLP provides the necessary non-linear capabilities, its fixed activation functions and relatively simple network structure may show limitations when dealing with complex or highly non-linear data patterns. Additionally, the standard MLP offers limited flexibility in parameter tuning and model scaling, which may restrict its effectiveness in various tasks and broader applications. Inspired by these insights, this paper replaces the MLP part of the PoolFormer architecture with a KAN. By incorporating KAN, we expect the model to maintain computational efficiency while more accurately capturing and representing complex data features.

The entire process of the KAN Transformer Block is illustrated in Figure 3d. The input image $I\in R^{H\times W\times C}$ first undergoes an input embedding:

(16) X=InputEmb(I)

where $X\in R^{N\times C}$ and N=H×W. This step serves to convert the input image *I* from its original spatial dimensions $R^{H\times W\times C}$ into a sequence of tokens *X*. Next, *X* is fed into repeated KAN Transformer Blocks, each of which contains two residual sub-blocks. Specifically, the first sub-block uses a pooling operation to act as a token mixer, and this process can be represented as

(17) Y=Pooling(Norm(X))+X

where $Norm\left(\cdot \right)$ normalization operations, such as layer normalization or batch normalization. The second residual sub-block replaces the original MLP with a two-layer structured KAN:

(18) $$Z=\left(\mathrm{Norm}\left(Y\right)\Phi_{1}\right)\Phi_{2}+Y$$

where $\Phi_{1}$ and $\Phi_{2}$ denote two layers of learnable activation functions within the KAN.

## 4. Experiments

In this section, we evaluated the proposed method on three fabric datasets. We began by introducing the datasets, selecting evaluation metrics, and describing the experimental details. Next, we conducted comparative experiments against several advanced models. Additionally, we performed ablation studies to further verify the effectiveness of our method. Finally, we conducted visualization experiments to more intuitively perceive the differences between the various methods.

### 4.1. Datasets

- ***Four-Fabric-Defects Dataset:*** Currently, there is no multi-class semantic segmentation dataset specifically for fabric defects. Therefore, we collect and annotate four types of fabric defects: Hole, Oil, Stain, and ThreadError. The four types of fabric defects consist of a total of 325 images, and image enhancement techniques such as rotation, scaling, and random cropping are used to expand the dataset to 1625 images.
- ***Fabric Dataset [38]:*** This dataset contains a total of 1600 fabric defect images, which only include defects and the background. The defects in the dataset have weak features and contain false defects, making the detection task quite challenging.
- ***ZJU-Leaper Dataset [3]:*** This is currently the largest fabric dataset. It consists of 15 types of fabric texture images, which are divided into 4 groups based on the complexity of the background texture. We randomly selected 500 defect images for each of the 15 types of fabric textures, resulting in a total of 7500 images to be used as the dataset.

The three datasets mentioned above all use images with a size of 512 × 512 pixels. The ratio of the training set to the test set is 4:1.

### 4.2. Implementation Details and Evaluation Metrics

- ***Implementation Details:*** The experiments in this paper are deployed on mmsegmentation [39]. The learning rate is set at 0.01, momentum is at 0.9, using the stochastic gradient descent algorithm, with a weight decay of 0.0005. The maximum number of iterations is set at 40,000, with a batch size of 8, on an RTX 4060Ti GPU.
- ***Evaluation Metrics:*** We use Pixel Accuracy (PA), Mean Intersection over Union (mIoU), and Mean Dice Coefficient (mDice) as evaluation metrics.

### 4.3. Comparative Experiments

To validate the effectiveness of the method proposed in this paper, we conducted comparative experiments with six advanced models: U-Net [20], PSPNet [21], DeepLabV3+ [23], Segformer [28], KNet [29], and TransNeXt [40]. The results and analysis of these experiments will be discussed in subsequent sections of the paper.

#### 4.3.1. Results of the Four-Fabric-Defects Dataset

The quantitative results of the Four-Fabric-Defects Dataset are presented in Table 1. Our method achieves 99.33% PA, 91.60% mIoU, and 95.46% mDice, outperforming all other models while maintaining competitive efficiency. Among the CNN-based methods, U-Net performs the worst, achieving only 70.59% mIoU with a relatively small parameter size (28.9 M) but a high computational cost (203 G Flops), indicating its limitations in handling multi-class fabric defect images. DeepLabV3+, the best-performing CNN-based model, achieves 89.59% mIoU and 94.32% mDice but requires 41.2 M parameters and 177 G Flops. Our approach surpasses it by 0.81% in mIoU and 1.14% in mDice, demonstrating a better trade-off between accuracy and computational efficiency. For Transformer-based architectures, KNet achieves the highest performance among its peers, outperforming all CNN-based models in PA, mIoU, and mDice. However, our method still exceeds KNet, with a 0.74% improvement in mIoU, with fewer parameters (37.8 M) and significantly lower computational cost (167 G Flops). These results highlight the superior capability of our method in addressing complex multi-class fabric defect images. By effectively combining the strengths of CNNs and Transformers, HKAN not only achieves SOTA performance but also provides a more efficient solution with reduced computational overhead and model size compared to other Transformer-based methods. This balance between accuracy, parameter efficiency, and computational cost makes HKAN particularly suitable for practical applications.

#### 4.3.2. Results of Fabric Dataset

The quantitative results of the Fabric Dataset are shown in Table 2. Our method achieves the highest performance in terms of mIoU and mDice, reaching 70.41% and 79.08%, respectively. For PA, our method achieves 99.68%, slightly trailing PSPNet and Segformer by only 0.02%. The weak defect features in this dataset, coupled with their low contrast against the background texture, result in generally low mIoU scores across all models. Moreover, the performance gap between CNN-based and Transformer-based methods is minimal, indicating that neither architecture alone is sufficient to fully address the challenges posed by this dataset. These results suggest that there is still considerable room for improvement in fabric defect segmentation, particularly in enhancing the ability to distinguish subtle defects from complex background textures.

#### 4.3.3. Results of ZJU-Leaper Dataset

The quantitative results for Group 1 and Group 2 in the ZJU-Leaper Dataset are shown in Table 3. Our method achieved the best results in both tissue datasets. In Group 1, the fabric background texture is single and clear, and the fabric defects are not complex, so the differences between the models are not easily noticeable, and all achieve good detection results. As the background texture becomes slightly more complex and the fabric defects become more diverse, the detection performance of different models shows significant differences. Our method achieved a mIoU in Group 2 that was 4.42% higher than UNet and 0.14% higher than the best-performing model, DeepLabV3+. In Groups 3 and 4 (Table 4), the fabric images contain complex fabric textures and diverse fabric defects. Our method achieved a PA in Group 3 that was only 0.01% lower than PSPNet, and the mDice reached 93.36% and 94.01% in Groups 3 and 4, outperforming other models. The experimental results indicate that our method can accurately capture image details when facing simple fabric defect images and still maintains strong competitiveness when dealing with complex fabric defect images. This is attributed to the KAN Transformer Block proposed in our method, which has the ability to handle long-distance dependencies.

#### 4.3.4. Visualization Results

**Figure 4:**
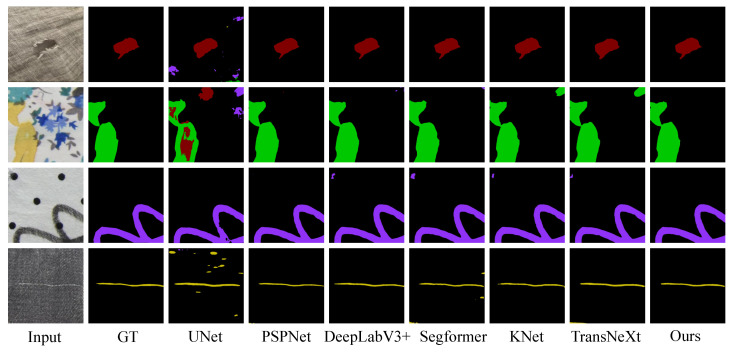
Intuitive visualization results of the Four-Fabric-Defects Dataset.

We displayed intuitive detection results of the Four-Fabric-Defects Dataset in Figure 4. From Figure 4, it is evident that for detecting simple Holes, most models perform well except for UNet. When detecting Oil, both KNet and TransNeXt suffer from false positives. In the case of Stain detection, DeepLabV3+, Sgeformer, and KNet all exhibit minor false positives in specific areas, whereas our method does not. Finally, the detection results of ThreadError show that our method provides clearer detection outcomes.

**Figure 5:**
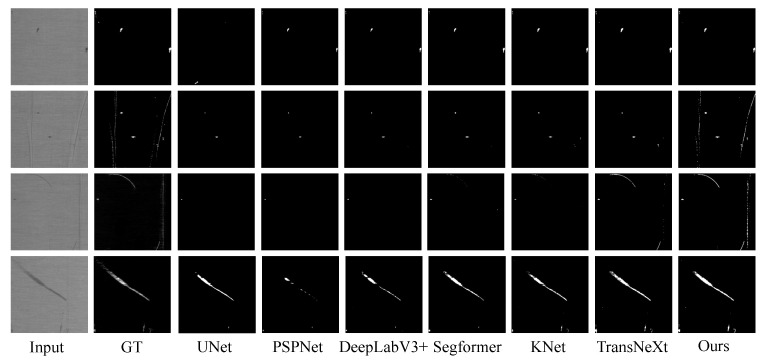
Intuitive visualization results of the Fabric Dataset.

Figure 5 shows the intuitive detection results of the Fabric Dataset. As can be seen from Figure 5, the detection performance of the three CNN-based models is weaker than that of the Transformer-based models. TransNeXt, which is a Transformer model, can detect the creases on the right side as shown in the third row of Figure 5, whereas Segformer and KNet cannot. Our method demonstrates superior detection performance compared to TransNeXt, with clearer and more complete boundaries. This can be observed more clearly in the second and third rows of Figure 5.

**Figure 6:**
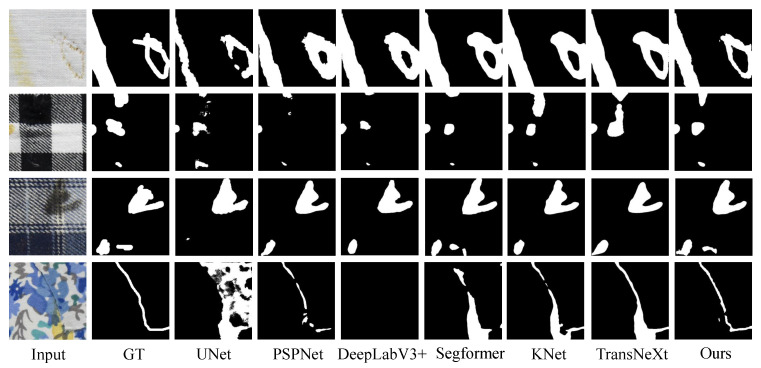
Intuitive visualization results of the ZJU-Leaper Dataset.

Figure 6 presents the intuitive detection results from our experiments on the ZJU-Leaper dataset. It is apparent from the image that our method significantly outperforms the compared models. Our model is able to accurately capture the boundaries of the targets, demonstrating excellent detail depiction capabilities even in complex scenes. In the presence of significant background interference, our model exhibits stronger robustness, effectively reducing areas of incorrect classification, while other models are somewhat lacking in this aspect. Additionally, our model performs more consistently in segmenting large-scale targets, mitigating issues such as fragmentation or insufficient coherence that are commonly observed in models like Segformer and KNet.

Across diverse scenarios, our approach adapts well to various inputs, consistently producing high-quality segmentation results.

### 4.4. Ablation Experiments

To further verify the effectiveness of the proposed module in this paper, we conducted ablation experiments on the Four-Fabric-Defects Dataset. We selected the UNet based on pure CNN as the baseline method and sequentially chose PoolFormer Block, KANConv Block, KANTransformer Block, and Hybrid KAN Block as the Encoders. The quantitative experimental results are shown in Table 5. From Table 5, it can be seen that replacing the convolutional modules in UNet with KANConv can significantly improve the detection performance, but there is still a certain gap with mainstream models. Using the PoolFormer Block and KAN Transformer Block as Encoders can further improve the detection performance of the model, but it still does not reach the level of SOTA models. Using the entire Hybrid KAN Block as the Encoder, the detection performance is close to that of Segformer and KNet, which perform better. From the above experimental results and analysis, it can be seen that the KANConv Block, KANTransformer Block, and Hybrid KAN Block proposed in this paper play different roles to collectively enhance the model’s feature extraction ability, fully combining the advantages of each model.

**Figure 7:**
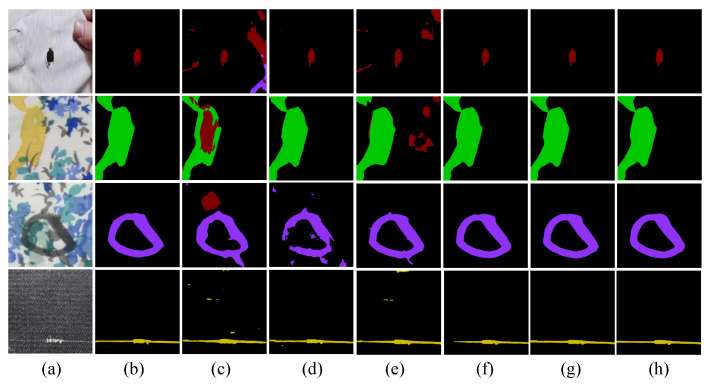
Ablation Experiment Detection Results. (**a**) Input. (**b**) Ground truth. (**c**) CNN Encoder. (**d**) Transformer Encoder. (**e**) KANConv Encoder. (**f**) KANTransformer Encoder. (**g**) Hybrid KAN Encoder. (**h**) HAKN.

Figure 7 shows the intuitive detection results of the ablation experiments. From (c) and (e) in Figure 7, it can be seen that both the CNN Encoder and the KANConv Encoder perform poorly when dealing with complex fabric defects, with instances of false detection and misjudgment. From (d), it is observed that the Transformer Encoder performs better than the aforementioned encoders but tends to over-detect background pixels, leading to less effective results compared to the KANTransformer Encoder. From (g) and (h), it is evident that the proposed Hybrid KAN Encoder achieves better feature extraction than the pure CNN or Transformer, balancing both local and global features while effectively eliminating interference from complex background textures.

## 5. Conclusions

This paper proposes a fabric defect segmentation network called HKAN, which integrates CNNs, Transformers, and KANs into a unified framework, demonstrating significant potential for the fabric defect segmentation task. Extensive experiments were conducted on three fabric datasets, and the results indicate that HKAN can achieve excellent segmentation outcomes for both simple and complex fabric images, outperforming models based solely on CNNs or Transformers.

Beyond fabric defect segmentation, HKAN demonstrates remarkable versatility and has the potential to address challenges in various fields that require precise segmentation and defect detection. For instance, in medical imaging, HKAN’s ability to integrate local and global features makes it well-suited for tasks such as tumor boundary detection, organ segmentation, and micro-lesion identification. Despite its promising performance, HKAN has certain limitations. One of the major challenges lies in its computational complexity. Integrating CNNs, Transformers, and KANs into a unified framework increases the size and inference time of the models, which may hinder its application in resource-constrained environments such as edge devices or real-time systems.

In the future, we will aim to develop HKAN into a backbone suitable for various downstream tasks in computer vision to explore its potential applications in other fields.

## Figures and Tables

**Table 1:** Quantitative Comparisons with SOTA methods for the Four-Fabric-Defects Dataset.

Method	PA (%)	mIoU (%)	mDice (%)	Params (M)	Flops (G)
UNet [20]	96.57	70.59	83.08	28.9	203
PSPNet [21]	98.94	87.60	93.20	46.6	179
DeepLabV3+ [23]	99.08	89.59	94.32	41.2	177
Segformer [28]	99.21	90.79	94.99	51.5	173
KNet [29]	99.26	90.86	95.01	72.1	249
TransNeXt [40]	99.16	89.87	94.46	47.9	152
HKAN	**99.33**	**91.60**	**95.46**	37.8	167

**Table 2:** Quantitative Comparisons to SOTA methods on the Fabric Dataset.

Method	PA (%)	mIoU (%)	mDice (%)
UNet [20]	99.67	64.14	72.16
PSPNet [21]	**99.70**	67.33	75.83
DeepLabV3+ [23]	99.08	68.24	76.82
Segformer [28]	**99.70**	69.74	78.38
KNet [29]	99.69	69.35	77.99
TransNeXt [40]	99.69	69.57	78.21
HKAN	99.68	**70.41**	**79.08**

**Table 3:** Quantitative comparison with the SOTA method on Group 1 and Group 2 of the ZJU-Leaper dataset.

Model	Group 1			Group 2		
	PA (%)	mIoU (%)	mDice (%)	PA (%)	mIoU (%)	mDice (%)
U-Net [20]	97.26	83.17	90.18	96.69	81.46	89.02
PSPNet [21]	97.32	84.81	91.30	97.11	83.32	90.30
DeepLabV3+ [23]	97.65	85.73	91.88	**97.53**	85.74	91.90
Segformer [28]	97.75	86.40	92.32	97.45	85.45	91.71
KNet [29]	97.73	86.49	92.38	97.47	85.42	91.69
TransNeXt [40]	97.57	85.45	91.71	97.18	83.92	90.71
HKAN	**97.77**	**86.63**	**92.46**	**97.53**	**85.88**	**91.99**

**Table 4:** Quantitative comparison with the SOTA method on Group 3 and Group 4 of the ZJU-Leaper dataset.

Model	Group 3			Group 4		
	PA (%)	mIoU (%)	mDice (%)	PA (%)	mIoU (%)	mDice (%)
U-Net [20]	97.60	82.57	89.72	96.21	80.47	88.36
PSPNet [21]	**98.44**	87.99	93.28	97.96	88.81	93.84
DeepLabV3+ [23]	98.33	87.43	92.94	97.91	88.67	93.75
Segformer [28]	98.39	87.67	93.09	97.89	88.44	93.61
KNet [29]	98.19	86.45	92.31	97.68	87.81	93.23
TransNeXt [40]	98.21	85.80	92.54	97.61	87.27	92.89
HKAN	98.43	**88.10**	**93.36**	**97.98**	**89.10**	**94.01**

**Table 5:** Ablation experiment results of the Four-Fabric-Defects Dataset.

Method	PA (%)	mIoU (%)	mDice (%)
CNN Encoder	96.57	70.59	83.08
Transformer Encoder	98.69	85.28	91.66
KANConv Encoder	98.09	81.60	89.15
KANTransformer Encoder	98.93	87.52	93.10
Hybrid KAN Encoder	99.26	90.84	95.02
HKAN	**99.33**	**91.60**	**95.46**

## Data Availability

The links to the Fabric Dataset and ZJU-Leaper Dataset used in the paper are https://pan.baidu.com/share/init?surl=nA2gIhL35iSsbMstkEugfg&pwd=0927 (accessed on 20 June 2024) and http://www.qaas.zju.edu.cn/zju-leaper/ (accessed on 20 June 2024). If you are interested in the Four-Fabric-Defects Dataset used in this paper, please send an email to ye-pei@outlook.com and state the purpose.

## References

[1] Ngan H.Y., Pang G.K., Yung N.H. (2011). Automated fabric defect detection—A review. Image Vis. Comput..

[2] Rasheed A., Zafar B., Rasheed A., Ali N., Sajid M., Dar S.H., Habib U., Shehryar T., Mahmood M.T. (2020). Fabric defect detection using computer vision techniques: A comprehensive review. Math. Probl. Eng..

[3] Zhang C., Feng S., Wang X., Wang Y. (2020). Zju-leaper: A benchmark dataset for fabric defect detection and a comparative study. IEEE Trans. Artif. Intell..

[4] Zhao S., Yin L., Zhang J., Wang J., Zhong R. (2020). Real-time fabric defect detection based on multi-scale convolutional neural network. IET Collab. Intell. Manuf..

[5] Huang Y., Jing J., Wang Z. (2021). Fabric defect segmentation method based on deep learning. IEEE Trans. Instrum. Meas..

[6] Koulali I., Eskil M.T. (2021). Unsupervised textile defect detection using convolutional neural networks. Appl. Soft Comput..

[7] Chen M., Yu L., Zhi C., Sun R., Zhu S., Gao Z., Ke Z., Zhu M., Zhang Y. (2022). Improved faster R-CNN for fabric defect detection based on Gabor filter with Genetic Algorithm optimization. Comput. Ind..

[8] Ren S., He K., Girshick R., Sun J. (2016). Faster R-CNN: Towards real-time object detection with region proposal networks. IEEE Trans. Pattern Anal. Mach. Intell..

[9] Lu B., Huang B. (2024). A texture-aware one-stage fabric defect detection network with adaptive feature fusion and multi-task training. J. Intell. Manuf..

[10] Vaswani A. Attention is all you need. Proceedings of the 31st Annual Conference on Neural Information Processing Systems (NIPS 2017).

[11] Dosovitskiy A. (2020). An image is worth 16x16 words: Transformers for image recognition at scale. arXiv.

[12] Liu Z., Lin Y., Cao Y., Hu H., Wei Y., Zhang Z., Lin S., Guo B. Swin transformer: Hierarchical vision transformer using shifted windows. Proceedings of the IEEE/CVF International Conference on Computer Vision.

[13] Qu H., Di L., Liang J., Liu H. (2023). U-SMR: U-SwinT & multi-residual network for fabric defect detection. Eng. Appl. Artif. Intell..

[14] He K., Zhang X., Ren S., Sun J. Deep residual learning for image recognition. Proceedings of the IEEE Conference on Computer Vision and Pattern Recognition.

[15] Xu H., Liu C., Duan S., Ren L., Cheng G., Hao B. (2023). A Fabric Defect Segmentation Model Based on Improved Swin-Unet with Gabor Filter. Appl. Sci..

[16] Cao H., Wang Y., Chen J., Jiang D., Zhang X., Tian Q., Wang M. (2022). Swin-unet: Unet-like pure transformer for medical image segmentation. Proceedings of the European Conference on Computer Vision.

[17] Hornik K., Stinchcombe M., White H. (1989). Multilayer feedforward networks are universal approximators. Neural Netw..

[18] Liu Z., Wang Y., Vaidya S., Ruehle F., Halverson J., Soljačić M., Hou T.Y., Tegmark M. (2024). Kan: Kolmogorov-arnold networks. arXiv.

[19] Long J., Shelhamer E., Darrell T. Fully convolutional networks for semantic segmentation. Proceedings of the IEEE Conference on Computer Vision and Pattern Recognition.

[20] Ronneberger O., Fischer P., Brox T. (2015). U-net: Convolutional networks for biomedical image segmentation. Proceedings of the Medical Image Computing and Computer-Assisted Intervention–MICCAI 2015: 18th International Conference.

[21] Zhao H., Shi J., Qi X., Wang X., Jia J. Pyramid scene parsing network. Proceedings of the IEEE Conference on Computer Vision and Pattern Recognition.

[22] Xiao T., Liu Y., Zhou B., Jiang Y., Sun J. Unified perceptual parsing for scene understanding. Proceedings of the European Conference on Computer Vision (ECCV).

[23] Chen L.C., Zhu Y., Papandreou G., Schroff F., Adam H. Encoder-decoder with atrous separable convolution for semantic image segmentation. Proceedings of the European Conference on Computer Vision (ECCV).

[24] Zhang H., Dana K., Shi J., Zhang Z., Wang X., Tyagi A., Agrawal A. Context encoding for semantic segmentation. Proceedings of the IEEE conference on Computer Vision and Pattern Recognition, Salt Lake City.

[25] Kirillov A., Girshick R., He K., Dollár P. Panoptic feature pyramid networks. Proceedings of the IEEE/CVF Conference on Computer Vision and Pattern Recognition.

[26] Zheng S., Lu J., Zhao H., Zhu X., Luo Z., Wang Y., Fu Y., Feng J., Xiang T., Torr P.H. Rethinking semantic segmentation from a sequence-to-sequence perspective with transformers. Proceedings of the IEEE/CVF Conference on Computer Vision and Pattern Recognition.

[27] Strudel R., Garcia R., Laptev I., Schmid C. Segmenter: Transformer for semantic segmentation. Proceedings of the IEEE/CVF International Conference on Computer Vision.

[28] Xie E., Wang W., Yu Z., Anandkumar A., Alvarez J.M., Luo P. (2021). SegFormer: Simple and efficient design for semantic segmentation with transformers. Adv. Neural Inf. Process. Syst..

[29] Zhang W., Pang J., Chen K., Loy C.C. (2021). K-net: Towards unified image segmentation. Adv. Neural Inf. Process. Syst..

[30] Cheng B., Schwing A., Kirillov A. (2021). Per-pixel classification is not all you need for semantic segmentation. Adv. Neural Inf. Process. Syst..

[31] Cheng B., Misra I., Schwing A.G., Kirillov A., Girdhar R. Masked-attention mask transformer for universal image segmentation. Proceedings of the IEEE/CVF Conference on Computer Vision and Pattern Recognition.

[32] Li C., Liu X., Li W., Wang C., Liu H., Liu Y., Chen Z., Yuan Y. (2024). U-kan makes strong backbone for medical image segmentation and generation. arXiv.

[33] Rege Cambrin D., Poeta E., Pastor E., Cerquitelli T., Baralis E., Garza P. (2024). KAN You See It? KANs and Sentinel for Effective and Explainable Crop Field Segmentation. arXiv.

[34] Bodner A.D., Tepsich A.S., Spolski J.N., Pourteau S. (2024). Convolutional Kolmogorov-Arnold Networks. arXiv.

[35] Yang X., Wang X. (2024). Kolmogorov-arnold transformer. arXiv.

[36] He Y., Xie Y., Yuan Z., Sun L. (2024). MLP-KAN: Unifying Deep Representation and Function Learning. arXiv.

[37] Yu W., Luo M., Zhou P., Si c., Zhou Y., Wang X. MetaFormer is Actually What You Need for Vision. Proceedings of the IEEE/CVF Conference on Computer Vision and Pattern Recognition.

[38] Wang J., Xu G., Li C., Gao G., Wu Q. (2022). Sddet: An enhanced encoder–decoder network with hierarchical supervision for surface defect detection. IEEE Sens. J..

[39] Contributors M. (2020). MMSegmentation: OpenMMLab Semantic Segmentation Toolbox and Benchmark. https://github.com/open-mmlab/mmsegmentation.

[40] Shi D. TransNeXt: Robust Foveal Visual Perception for Vision Transformers. Proceedings of the IEEE/CVF Conference on Computer Vision and Pattern Recognition.

